# Physical Activity in Patients with Advanced Cancer: Sociodemographic, Clinical, and Psychological Correlates

**DOI:** 10.3390/brainsci14060573

**Published:** 2024-06-03

**Authors:** Luka Mihic-Góngora, Paula Jimenez-Fonseca, Sara Coca-Membribes, Patricia Cruz-Castellanos, Rocío Galán-Moral, Elena Asensio-Martínez, María Palacín-Lois, Alberto Carmona-Bayonas, Cristina Caramés-Sánchez, Caterina Calderon

**Affiliations:** 1 Department of Medical Oncology, Hospital Universitario Central de Asturias, 33007 Oviedo, Spain; 2 Department of Medical Oncology, Hospital Universitario de Canarias, La Laguna, 38320 Santa Cruz de Tenerife, Spain; 3 Department of Medical Oncology, Hospital General Universitario de Ciudad Real, 13005 Ciudad Real, Spain; 4 Department of Medical Oncology, Hospital General Universitario de Elche, 03203 Alicante, Spain; 5 Department of Clinical Psychology and Psychobiology, University of Barcelona, 08007 Barcelona, Spain; 6 Department of Medical Oncology, Hospital General Universitario Morales Meseguer, University of Murcia, IMIB, 30008 Murcia, Spain; 7 Department of Medical Oncology, Hospital Universitario Fundación Jiménez-Diaz, 28040 Madrid, Spain

**Keywords:** physical activity, advanced cancer, emotional distress, functional status, symptoms, quality of life

## Abstract

As cancer progresses, patients may experience physical decline, which can impair their ability to carry out essential daily tasks. The aim of this study was to analyze the levels of physical activity in patients with advanced cancer undergoing systemic treatment and its relationship with sociodemographic, clinical, and psychological factors. A prospective, cross-sectional, multicenter study was carried out in 15 oncology departments in Spain. Patients with locally advanced, unresectable, or metastatic cancer who were candidates for systemic treatment were included. Participants completed demographic information and psychological scales. In total, 508 patients were included in the study, the majority of whom were male, over the age of 65, and diagnosed with bronchopulmonary tumors (36%) and metastatic disease. Based on their physical activity levels, participants were categorized as sedentary (20%, *n* = 190), engaging in light physical activity (43%, *n* = 412), or demonstrating moderate physical activity (37%, *n* = 351). Patients who were over 65 years old; had a worse baseline status (ECOG ≥ 1); lacked a partner; had a lower educational level; or were retired or unemployed were found to have lower levels of physical activity. Those with sedentary physical activity reported higher levels of psychological distress, anxiety, depression, somatization, and physical symptoms, as well as worse functional status, global health status, and well-being. Understanding the complex interplay between physical activity and sociodemographic, clinical, and psychological factors can help neuroscientists develop tailored exercise interventions that address the unique needs of advanced cancer patients.

## 1. Introduction

In Europe, cancer is responsible for 1.9 million deaths every year [1]. Although these figures are concerning, medical advances made in recent years have led to longer survival periods in patients with advanced and incurable cancer [2]. However, the quality of life of these patients remains a significant challenge in cancer treatment. Weakness and fatigue are the most frequent symptoms in patients with advanced cancer, affecting 83% of patients [3]. In addition, a high percentage of patients suffer from a heavy symptom burden, including pain (64%), difficulty swallowing (84%), diarrhea (54%), anorexia (34%), and constipation (32%) [3,4].

Regular physical activity can improve many of the negative consequences reported by adults diagnosed with cancer [5,6]. Numerous studies and meta-analyses have confirmed the effectiveness of physical activity in reducing fatigue and increasing physical functioning in patients during adjuvant treatment [7,8,9]. The latest guidelines from the American Cancer Society recommend that patients with advanced stage tumors engage in physical activity [10]. This activity should consist of at least 150 min per week of moderate intensity or several bouts of at least 10 min each, and these recommendations should be adapted to the individual physical abilities of each patient [10]. Overall, the recommendation of physical activity for cancer patients is based on the benefits that such activity can provide, namely improvement in cardiovascular function, reduction of the risk of depression, and enhancement of quality of life [10]. However, despite the scientific evidence, less than 30% of patients currently meet these recommendations [11,12,13]. Studies that have examined the role of exercise in prostate and lung cancer patients have found that patients who engage in less physical activity exhibit greater weakness and depression compared to healthy individuals who exercise regularly [13,14]. It has also been observed that, in lung cancer patients, those who engage in less physical have poorer physical and mental health [15].

The integration of physical activity and palliative care can significantly improve the quality of life of patients with advanced cancer [5,15,16]. Physical activity can improve cardiorespiratory fitness, bone density, and reduce physical and functional symptoms [8,17]. Additionally, physical activity can relieve fatigue, pain, and dyspnea related to the tumor, prevent muscle loss during active treatment, and improve patients’ quality of life and physical fitness [11,18]. During oncological treatment, exercise can reduce treatment-related side effects and decrease symptom expression [15,19]. Physical activity can have positive effects on patients’ psychosocial health, such as reducing symptoms of anxiety and depression [5,16]. However, most research on the therapeutic effects of exercise has focused on patients in early stages of the disease, resulting in a significant research gap for patients with advanced cancer [20,21]. A systematic review of 16 studies concludes that physical activity is a safe and feasible intervention in patients with advanced cancer, improving overall health, symptoms, and quality of life [12]. In Germany, a study involving patients with metastatic cancer revealed that 32.0% participated in physical activities. Motivation was identified as a facilitator, while weakness, physical symptoms, depression, and anxiety were the main barriers [12]. Therefore, the present study aims to analyze the practice of physical exercise in patients with advanced cancer undergoing systemic treatment in Spain and its correlation with sociodemographic, clinical, and psychological factors—not only psychological distress and quality of life, but also well-being and the patient–doctor relationship—following diagnosis and prior to the initiation of systemic antineoplastic treatment [22].

## 2. Materials and Methods

### 2.1. Participants and Procedures

**Figure 1:**
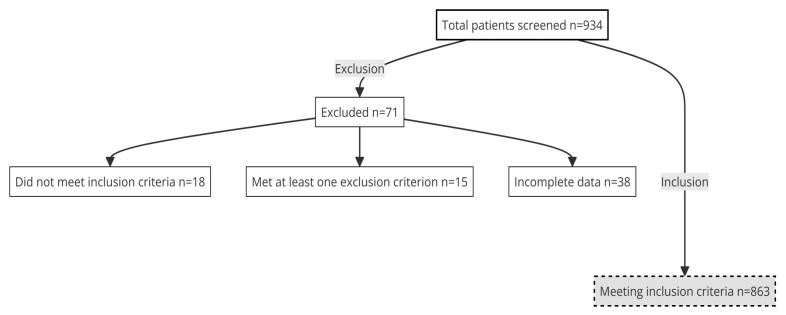
Patient recruitment.

This study was designed as a multicenter, prospective data collection study with a cross-sectional design. Between February 2020 and December 2022, a consecutive sample of advanced cancer patients was recruited from 15 medical oncology departments across different hospitals in Spain. Patients were recruited during their initial visit to the medical oncologist, where they were informed about their diagnosis, stage, incurable status, and therapeutic plan. To be candidates for inclusion in the study, patients had to be ≥18 years old, have histologically confirmed advanced cancer, and not be candidates for curative therapy or surgery. Exclusion criteria comprised of having a physical condition, comorbidity and/or age that contraindicated antineoplastic treatment; having received treatment for another advanced malignancy within the last two years; or having any additional personal, familial, sociological and/or medical condition that could impede participation in the study. Also, patients with cognitive impairment or severe deterioration of performance status due to cancer or other causes were excluded. The study received approval from the Ethics Review Committees of each participating institution and from the Spanish Agency of Medicines and Health Products (AEMPS; identification code: ES14042015), and was conducted in accordance with ethical principles. Participants completed questionnaires and provided clinical data, which were collected through interviews and medical records. Data collection procedures were standardized across all hospitals, and patient data were obtained from the institutions where they received treatment. Participation was voluntary and anonymous, and it did not affect patient care. All participants provided informed consent before being included in the study, which was delivered by the medical oncologist. Data were collected and updated by the medical oncologist through a web-based platform: www.neoetic.es (accessed on 10 January 2023). A total of 934 patients were screened, with 863 meeting the inclusion criteria and 71 being excluded (18 did not meet the inclusion criteria, 15 met at least one exclusion criterion, and 38 had incomplete data at the time of analysis); see Figure 1.

### 2.2. Measures

Patients reported their demographic information, such as age, sex, marital status, and whether they had children, and educational level in writing. They also completed five questionnaires (GSLTPAG, BSI, EORTC, WHO, and STAR) at home during the period between their first visit to the oncologist and the commencement of systemic treatment. The medical oncologist collected clinical variables related to cancer, antineoplastic treatment, and outcomes (including estimated survival) from the patients’ medical records.

Their level of physical activity was measured using a modified version of the Godin–Shepard Leisure-Time Physical Activity Questionnaire (GSLTPAQ) [23], assessing whether patients engaged in physical activity (no activity, light, moderate, or vigorous activity). The frequency with which they engaged in physical activity (less than once a month, 3–5 times a month, 1–2 times a week, 3–5 times a week) and for how long (less than 10 min, 20–30 min, more than 30 min) they exercised. This scale was used in patients with breast, prostate, and colorectal cancer patients [24].

The Brief Symptom Inventory (BSI) is a widely used tool for evaluating psychological distress [25]. It comprises 18 descriptions of physical and emotional symptoms categorized into three dimensions: anxiety, depression, and somatization. The anxiety subscale measures symptoms such as nervousness, tension, motor restlessness, apprehension, and panic states, while the depression subscale evaluates symptoms of dysphoric mood, disaffection, self-deprecation, anhedonia, hopelessness, and suicidal ideation. Each item is scored on a 5-point Likert scale, and the subscale score ranges from 0 to 24, with higher scores indicating greater anxiety or depression. Raw scores are converted to T-scores using gender-specific normative data. The BSI uses the clinical case rule [25]. The Spanish version of the BSI has demonstrated good reliability and validity in Spanish patients [25], with Cronbach’s alpha scores for the subscales ranging from 0.75 to 0.88.

The WHO-5 (Well-Being) questionnaire consists of 5 items and is rated on a 6-point scale ranging from 0 to 5. The total score ranges from 0 to 25, with higher scores indicating a better sense of well-being. The WHO-5 has been shown to have good internal reliability and construct validity [26], and it has been widely used in Spanish populations.

The evaluation of the doctor–patient relationship was conducted using the Therapeutic Relationship Assessment Scale (STAR-P), developed to assess the therapeutic alliance between patients and their clinicians [27]. It contains 12 items, each of which is assessed on a 5-point Likert scale ranging from 0 (never) to 4 (always). A greater score on the scale suggests a better patient–doctor relationship. It should be noted that the internal consistency of the total scores is robust, as shown by previous research [27], a finding that is confirmed in our study sample (Cronbach’s α = 0.85).

### 2.3. Data Analysis

Demographic and other variables were reported as mean, standard deviation (SD), number (N), and percentage (%) as appropriate for descriptive statistics. To identify patients with similar physical activity patterns, a cluster analysis was conducted using the GSLTPAG items as clustering variables. Participants with any missing GSLTPAG values were excluded from the final sample of n = 863 used for the cluster analysis. The k-means method with Euclidean distances between observations and Ward’s hierarchical clustering method were used to estimate clusters based on the squared error criterion [28,29,30]. Chi-square analyses evaluated differences in demographic, clinical, and psychological characteristics among the physical activity profiles, while ANOVA was used to assess differences in psychological characteristics. Post hoc contrasts were made using Bonferroni correction. Effect size was quantified using Eta squared (η2), with η^2^ between 0.01 and 0.06 indicating a small effect, η^2^ between 0.07 and 0.14 indicating a medium effect, and η^2^ greater than 0.14 indicating a large effect size [31]. The data were analyzed using the Statistical Package for Social Sciences (SPSS) for Windows 26.0 (SPSS Inc., Chicago, IL, USA), with a significance level of *p* < 0.05.

## 3. Results

### 3.1. Sociodemographic and Clinical Characteristics

Data from 863 participants (mean age, 65 ± 11) were included in the analysis after excluding missing data (90% response rate). Thirty-two physicians from 15 hospitals all over Spain participated in recruitment. Of the participants, 55% were male, 68% were married, 46% had completed junior high school, and 50% were either retired or unemployed. The most prevalent types of tumors were bronchopulmonary (29%), colorectal (16%), and pancreas (9%), with adenocarcinoma being the most common histology (65%). The majority of cancers were diagnosed at stage IV (80%). Chemotherapy alone or combined with other treatment modalities was the most frequent form of treatment. The estimated survival time was less than 18 months for 45% of the sample (refer to Table 1 for details).

### 3.2. Physical Activity Profiles and Clinical-Demographic Characteristics

In order to identify distinct physical activity profiles among the study population, we employed a k-means method using Euclidean distances and Ward’s hierarchical clustering method. Based on the results of the GSLTPAG scale and cluster analysis, participants were classified into three groups: those who were insufficiently active/sedentary (20%, n = 190), those with light physical activity (43%, n = 412), and those with moderate physical activity (37%, n = 351). Walking, slow-paced walking, and gardening were the physical activities most frequently performed (43%) by the patients, while fast-paced walking, going to the gym, or cycling were reported by 30% of the sample, and only 7% reported doing intensive physical activity.

**Figure 2:**
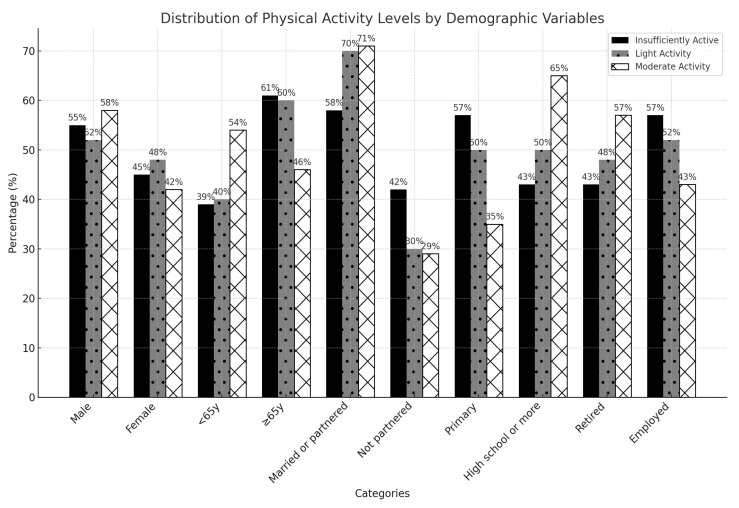
Demographic characteristics of patients by physical activity level (*n* = 863).

To examine the association between physical activity profiles and clinical and demographic characteristics, we categorized the Godin questionnaire responses dichotomously and conducted further analyses to state that certain categories are associated with more or less physical activity. We found that individuals aged 65 years or older (*X*^2^ = 18.640, *p* = 0.001); not partnered (*X*^2^ = 11.091, *p* = 0.004); primary-level education (*X*^2^ = 30.087, *p* = 0.001); and retired or unemployed (*X*^2^ = 11.392, *p* = 0.001), were more likely to practice physical activity than those individuals aged 65 years old or less; married; with high school-level education; and with employment, see Figure 2.

In relation to clinical characteristics, individuals with adenocarcinoma (*p* = 0.038), better baseline status (ECOG = 0) (*p* = 0.001), and estimated survival more than 18 months (*p* = 0.001) had a higher probability of practicing physical activity, see Table 1.

### 3.3. Physical Activity Profiles and Psychosocial Characteristics

In terms of the EORTC-QLQ-C30 scale, patients who were insufficiently active/sedentary displayed clinically significant worse scores on physical function (*p* = 0.001; η^2^ = 0.177); role function (*p* = 0.001, η^2^ = 0.080); cognitive function (*p* = 0.001, η^2^ = 0.021); emotional function (*p* = 0.001; η^2^ = 0.017); and social function (*p* = 0.001, η^2^ = 0.021), and more symptoms on fatigue (*p* = 0.001, η^2^= 0.033); nausea (*p* = 0 0.001, η^2^ = 0.16); pain (*p* = 0.001, η^2^ = 0.033); dyspnea (*p* = 0.001, η^2^ = 0.015); insomnia (*p*= 0.001, η^2^ = 0.010); appetite loss (*p* = 0.001, η^2^ = 0.039); constipation (*p* = 0.001, η^2^ = 0.016); and diarrhea (*p* = 0.001, η^2^= 0.014).

In general, patients who were insufficiently activity/sedentary displayed worse functional status (*p* = 0.001, η^2^ = 0.081) and global health status (*p* = 0.001, η^2^ = 0.014), and more symptoms (*p* = 0.001, η^2^ = 0.058) than patients with light and moderate physical activity. The BSI-18 scale revealed that patients who were insufficiently activity/sedentary presented more depression symptoms (*p* = 0.001, η^2^ = 0.019); anxiety (*p* = 0.014, η^2^ = 0.009); and somatization (*p* = 0.001, η^2^ = 0.048), and more psychological distress (*p* = 0 0.001, η^2^ = 0.026) and worse well-being (*p* = 0.007, η^2^ = 0.045) than patients with light and moderate physical activity. Relationship with physician scores were no different between patients in the three classes (see Table 2).

## 4. Discussion

The study’s primary objective was to investigate the levels of physical activity in advanced cancer patients prior to the initiation of systemic antineoplastic treatment, and their relationship to demographic, clinical, and psychological factors, including psychological distress, quality of life, well-being, and the patient–doctor relationship. This analysis has been conducted in a broad sample, and the combination of all variables and questionnaires applied together has not been reported in previous studies.

In our series, we found that 20% of patients with advanced cancer reported a lack of physical activity and a predominantly sedentary lifestyle, in which they spend most of their day sitting or lying down with little or no physical activity. A total of 43% of the sample reported engaging in physical activities that involve minimal effort, such as brisk walking, doing household chores, or gardening, while 37% of patients reported engaging in physical activities that require moderate effort, such as running, swimming, cycling, or going to the gym [20,32,33]. In various studies, it has been indicated that active involvement in physical activity is around 50–60% among cancer patients [5,32,33]. The main reason for not exercising in patients with advanced cancer was health-related deterioration or disease progression [11,12,20,32]. The majority of studies indicate that physical activity improves the quality of life and emotional well-being of patients [11,18,32], with only two indicating adverse events in 2% of patients. The main adverse events related to physical activity were muscular discomfort, cardiac complaints, and increased fatigue [9,15]. In those studies where, physical activity was incorporated as an intervention, the recruitment rate was around 38% in patients with colorectal cancer [6] and 59% in patients with lung cancer [19]. In our series, walking was the preferred form of physical activity for 80% of participants, similar to what was found in other studies where patients preferred walking [16].

Younger individuals, and those with a partner, higher educational level, and employment were more likely to engage in physical activity than older individuals; those without a partner; with primary education; and retired, or unemployed individuals who led a more sedentary lifestyle. As people age, their physical faculties progressively decline and they become more dependent on caretakers for everyday tasks. In addition, new comorbidities arise that make them more fragile and prone to serious illnesses [5,34]. Our research revealed that patients with lower performance status (ECOG > 1) and lower estimated 18-month survival exhibited decreased levels of physical activity. The physician’s estimate of baseline functional status correlated closely with the patients’ perception of their physical activity. Furthermore, self-perceived patient decline was associated with lower physical activity. In a study conducted with patients with advanced cancer and worse perceived health status, the majority of patients expressed the need to engage in physical activity to feel better [16,35]. A total of 47% preferred to do it in a hospital setting and 36% preferred to split the location of physical activity between home and hospital [20,21]. However, studies show that 84% of participants prefer to engage in physical activity at home. In contrast, it is to be noted that no existing study has reviewed physical activity profiles based on predicted survival at the time of diagnosis of advanced cancer and before the initiation of treatment [5]. The results of our study may be justified by the correlation between a lower survival estimate (less than 18 months) and clinical deterioration, changes in the individual’s surroundings, loss of independence, and emotional shifts due to fear of impending death, all of which may lead to a diminished level of physical activity. Consistent with this, our results suggest that the estimation of prognosis at the time of diagnosis is a significant risk factor for a decrease in physical activity.

In our series, patients who engaged in some form of physical activity had better functional status, fewer symptoms, better quality of life, less psychological distress, and greater well-being. Consistent with other studies, patients with advanced cancer who engage in physical activity improve their functional status [5,32,35]. In a study in which a group physical activity program was implemented for patients, significant improvements were observed in functional and social roles [21]. However, not all studies find statistically significant differences. Some studies find a trend towards improvement without reaching significance between physical activity and functional status [5,35], and there are those who have not found differences in functional well-being in patients with advanced cancer [36]. This may be due to the fact that these are very fragile patients, and depending on the samples analyzed, they may be patients with significant functional deterioration related to treatment.

Regarding symptoms, patients with advanced cancer often experience multiple concurrent symptoms that are harmful or debilitating and appear as a result of treatment and the disease process [37]. In our series, patients who reported engaging in more physical activity had fewer symptoms than those who did not engage in physical activity. Specifically, improvements were observed in the reduction of fatigue, pain, and nausea, as well as in the improvement in appetite. Among patients with advanced cancer, fatigue is not only the most common symptom, but also the most disruptive and longest-lasting. Studies suggest that 60 to 90% of patients with advanced cancer experience fatigue [38,39], as well as other symptoms like pain, nausea, and dyspnea [39,40]. Consistent with other research, patients who engage in physical activity during treatment improve fatigue and decrease pain [5,12,35]. The performance of physical activity in patients in a palliative care setting improves pain, reduces fatigue, and improves appetite. Most studies indicate that there is an improvement in physical symptoms with the practice of physical activity, especially in the reduction of fatigue and pain, which are two of the most common symptoms that affect activity levels [4,5,35]. This is especially relevant when we consider that cancer-related fatigue negatively affects quality of life [39,41]. Fatigue alters different biological pathways during the course of cancer, such as muscle metabolism or proinflammatory cytokines due to the tumor itself or as a side effect of treatment [16,42]. Physical activity can reverse this biological process and alleviate the expression of different biological pathways, improving patients’ quality of life [43].

In our case series, it was found that the practice of physical activity was associated with fewer symptoms of anxiety, depression, somatization, and psychological distress, and more emotional well-being. These findings are consistent with other studies indicating that physical activity during oncologic treatment can reduce its side effects and decrease anxiety and depression [19,44].

Although most research on the therapeutic effects of physical activity has been carried out during active treatment of early-stage cancer or during survivorship, there is still no standard recommendation for patients with incurable cancer [17,45]. However, it appears that physical activity can improve the emotional state of these patients [5,19]. The relevance of this study lies in its potential to support medical professionals, physiotherapists, and physical activity therapists in developing evidence-based solutions to reduce symptom burden in advanced cancer patients through the recommendation of physical activity.

### Limitations

Our study has several strengths, including access to sociodemographic and clinical information from a large representative sample across multiple regions, and the linking of participant and physician data. However, our study also has certain limitations. Firstly, it is cross-sectional in nature, making it impossible to determine the direction of observed relationships. Future longitudinal cohort studies are required to validate the findings from this study. Secondly, we relied on self-reported measures, which may introduce response bias, such as social desirability or memory errors. Lastly, our results are not generalizable to patients with resectable cancer.

## 5. Conclusions

Engaging in physical activity can benefit both patients with advanced cancer and their families. Routine assessment of patients’ activity patterns, along with advice, counseling, and encouragement related to maintaining or increasing physical activity levels in patients with cancer is likely to be important. Improving, delaying deterioration, or maintaining functional status, symptom management, quality of life, and emotional state for patients with advanced cancer through physical activity has the potential to reduce caregiver burden, as well as the medical costs associated with longer hospital and specialty center stays. Therefore, support and research into physical activity practice and the development of personalized programs for patients with advanced cancer can be an important method for potentially reducing some common side effects of cancer and its treatment, and improving patients’ quality of life.

## Figures and Tables

**Table 1:** Differences in clinical characteristics of patients by physical activity level (n = 863).

Variables	Total Sample n (%) 863 (100%)	Insufficiently Active/Sedentary n = 190 (20%)	Light Physical Activity n = 412 (43%)	Moderate Physical Activity n = 351 (37%)	*X* ^2^	*p* Value
Tumor site					11.810	0.298
Bronco-pulmonary	280 (29)	65 (34)	123 (30)	92 (26)		
Colorectal	154 (16)	23 (12)	65 (16)	66 (19)		
Pancreas	88 (9)	17 (9)	45 (11)	26 (7)		
Breast	78 (8)	12 (6)	34 (8)	32 (9)		
Stomach	48 (5)	8 (4)	23 (6)	17 (5)		
Others	305 (32)	65 (34)	122 (30)	118 (34)		
Histology					10.456	0.005
Adenocarcinoma	616 (65)	104 (55)	273 (66)	239 (68)		
Others	337 (35)	86 (45)	139 (34)	112 (32)		
Stage					6.562	0.038
Locally Advanced	189 (20)	50 (26)	78 (19)	61 (17)		
Metastatic Dis. (IV)	764 (80)	140 (74)	334 (81)	290 (83)		
Type of treatment					8.838	0.183
Chemotherapy	521 (55)	118 (62)	227 (55)	176 (50)		
Chemo+ Immuno	220 (23)	39 (21)	94 (23)	87 (25)		
Chemo+ Antidian	195 (20)	32 (17)	82 (20)	81 (23)		
Others	17 (2)	1 (1)	9 (2)	7 (2)		
ECOG					40.954	0.001
0	360 (38)	48 (25)	135 (33)	177 (50)		
1 or more	593 (62)	142 (75)	277 (67)	174 (50)		
Survival					19.903	0.001
<18 months	425 (45)	104 (55)	195 (47)	126 (36)		
≥18 months	524 (55)	86 (45)	217 (53)	225 (64)		

**Table 2:** Psychosocial characteristics of physical activity profile.

	Insufficiently Activity/Sedentary n = 190		Light Phys. Activity n = 412		Moderate Phys. Activity n = 351				
	Mean	SD	Mean	SD	Mean	SD	F	*p*	η^2^
EORTC-QLQ-C30									
Physical function	55.6	28.9	68.0	25.0	85.4	19.0	101.32	0.001	0.177
Role function	58.5	38.1	63.3	33.9	80.4	27.0	40.915	0.001	0.080
Cognitive function	71.5	29.4	78.2	25.3	81.3	23.8	10.157	0.001	0.021
Emotional function	56.6	31.6	62.0	29.1	65.9	29.3	8.151	0.001	0.017
Social function	34.5	37.1	64.3	34.3	72.6	31.9	10.136	0.001	0.021
Fatigue symptoms	58.5	32.1	50.2	29.8	35.2	30.2	45.956	0.001	0.089
Nausea/vomit	22.5	32.5	16.1	27.0	13.1	33.4	7.637	0.001	0.016
Pain symptoms	44.5	37.6	37.8	33.5	29.9	33.4	16.041	0.001	0.033
Dyspnea	17.7	32.4	14.3	27.2	9.1	22.4	7.257	0.001	0.015
Insomnia	50.0	37.8	44.5	37.3	41.2	38.4	4.667	0.010	0.010
Appetite loss	44.7	42.1	37.0	38.6	24.1	34.7	19.171	0.001	0.039
Constipation	44.9	42.3	33.7	38.0	31.9	38.0	7.691	0.001	0.016
Diarrhea	27.7	37.8	20.7	33.5	17.6	31.6	6.887	0.001	0.014
Financial difficulties	20.7	33.3	18.7	32.2	19.1	33.0	1.653	0.192	--
Functional status	61.4	25.3	67.2	22.3	77.1	19.6	41.528	0.001	0.081
Symptom status	36.8	21.5	30.3	18.9	24.6	19.8	29.243	0.001	0.058
Global health status	58.9	25.1	58.0	23.6	64.4	25.4	6.780	0.001	0.014
Brief Symptom Inventory									
Depression	64.0	7.6	63.8	7.3	62.1	6.5	9.072	0.001	0.019
Anxiety	65.4	8.5	65.0	8.2	63.9	7.8	4.322	0.014	0.009
Somatization	67.4	8.8	65.9	7.9	63.2	7.4	23.649	0.001	0.048
Psychological distress	68.4	8.1	68.0	7.5	66.0	7.1	12.308	0.001	0.026
Well-being (WHO)	16.7	5.4	16.2	4.8	18.5	4.7	22.188	0.007	0.045
Relationship with physician	38.6	7.2	38.6	7.5	39.7	6.7	2.673	0.070	--

Abbreviation: EORTC-QLQ-C30, European Organization for Research and Treatment of Cancer Quality of Life Questionnaire; WHO, Well-Being.

## Data Availability

The datasets generated and analyzed during the current study are not publicly available for reasons of privacy. They are however available (fully anonymized) from the corresponding author on reasonable request.
